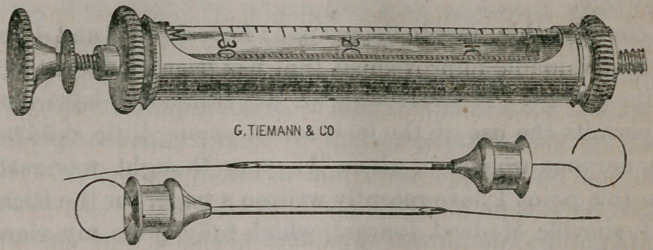# Description of a New Pocket Case for Physicians

**Published:** 1875-12

**Authors:** William A. Greene

**Affiliations:** Americus, Georgia


### DESCRIPTION OF A NEW POCKET CASE FOR PHYSICIANS.

### Containing a New Hypodermic Syringe and Needle, with a few Remarks Concerning the use of the Latter.

By WILLIAM A. GREENE, M.D., of Americus, Ga.

I have recently devised a Pocket Medical Case, which I consider far superior to anything of the kind hitherto manufactured. A brief description of it will readily show its advantages and convenience. The case is six inches long, three inches wide, and two and one-half inches thick, made of the best material, and lined with blue silk velvet, presenting a very neat appearance and easily carried in the pocket.

It has a lower and upper department, opening in the center, and containing equal space for its contents. In the lid, or upper department, you have, first, a pocket thermometer, self-registering, contained in a strong and neat vulcanized rubber case, perfectly correct and warranted in all respects. Every intelligent physician of the day confesses this instrument almost indispensable, and should always be in hand. Next, a hypodermic syringe of the most approved and modern manufacture; in fact, the best in use and only one worth having, and I think has decided advantages over others, which consist in the peculiar construction of this instrument. It has a nickle-plated encasement, fenestrated on both sides, on which the taps at both ends screw, contained in which is the glass cylinder accurately registered for thirty minims, and so arranged with washers at both ends in contact with this glass cylinder as, when the taps are screwed on closely, it is impossible to leak, or for a particle of the fluid to escape, except through the needle or point. Every one knows that those fastened with sealing-wax, cement, or anything of the kind, are constantly getting out of fix, and consequently very annoying—especially when a patient is *suffering* and pleading for *relief.* Many, no doubt, are the "cuss words" that have escaped the lips of the unchanged doctor, and profane thoughts that have traversed the brain of the more settled and would-be pious M.D. on this account. The double fenestratem admits the light from both sides, and you have no trouble in seeing what you are doing, either with dim gas or dim lights. While these are great advantages, I have recently devised still greater, as every patient will testify who, unfortunately, requires to be punctured, and their name is legion. I refer to the point, or needle, which should always be made to *slide on*, not *screwed on*, as many do, as the latter become loose by the threads of the screw wearing out, both from constant use and their destruction from coming in contact with acid or other solutions used. The former, the more they are used the *tighter* they fit. These points are made very *small* and *delicate*. In fact, the Messrs. Teiman, after repeated efforts and failures, succeeded in getting up a needle only a little larger than the proboscis of a fly. I assert *most positively* that the introduction of this needle, with a sol. sulph morph., twenty grains to the ounce, (which requires no acid to dissolve,) is without pain, and I have administered to little children without their knowledge.

I consider this, next to the syringe I have devised and described, the most valuable improvement that has been made in the hypodermic syringe. It overcomes an impediment I have long felt, and permits the use of the instrument among little children, and those nervous women in whom the very thought was revolting. Upon this point I have recently written a paper for the Richmond and Louisville Medical Journal, which fully gives my views and experience with reference to the hypodermic administration of remedies to children, and which I presume will soon be published. Also, a full description of these delicate needles and their use, which I hope all physicians will read, criticise, and give their experience to the profession.

The case further contains extra wires and washers, the former for cleansing the points and the latter for putting on when required, by simply unscrewing the nut through which the piston plays—the piston being made longer than the cylinder to allow the washer being pushed through far enough for this purpose, or to be oiled, without the trouble of getting it out in the old way. There is also a very fine hone, or fine stone, for sharpening the points, which is frequently needed; also, a few pieces of emory paper for smoothing the needles, etc. Every physician knows how often his needles require to be sharpened, and how difficult it is to procure a suitable stone to sharpen them on. There is also a twenty-four minim measure, very accurate. There are five two drachm vials, (empty,) to be filled as each physician may fancy. I fill two of them with solutions of sul. morphia, one with varatrum viride (Norwood's) one with sol. sulph. atropa, and the fifth with alcohol for cleaning the points; and the powder department to be filled as the physician may desire. I have also spoken freely of this in the above mentioned paper, written for the Richmond and Louisville Medical Journal. The lower department, or body of the case, contains four four-drachm vials for medicines, a place for two hypodermic needles, and a *thumb lancet* of the best manufacture.

In this connection I am reminded of the valuable and able paper on "blood-letting," read before the Georgia State Medical Association, at Savannah, last April, by that glorious gentleman and polished physician, Dr. C. B. Nottingham, of Macon, Ga., and I wish it was engraved upon every *lancet* that is manufactured in the land, and stamped indelibly upon the memory of every physician; then might we return to a long neglected therapeutic agent, than which, in my humble opinion, there is none more valuable and efficacious in the annals of medicine. Long may he live to contribute from his almost inexhaustable store of information and experience, yet more of that priceless knowledge that a long life of laborious research and close application has made him master of. What a boon to mankind is such a physician; almost noiseless in his daily rounds, gathering and culling knowledge in the silent hours of the night, at the bedside of the most miserable pauper, without fee or reward, and none to praise or herald his triumphant success over loathsome disease; only the gentle workings of a conscience that tells of duty well performed, or gladdens his wearied mind with fresh discoveries, which open up new fields for research and study, from whence he may gather yet more valuable material to lavishly apply with unsparing hand upon every one who seeks relief at his door to stay the inroads of some fatal malady threatening the brittle thread of life, be they some miserable Lazarus or the "rich man clothed in purple and fine linen and faring sumptuously every day." Such is the life of the true physician, and high upon this pinnacle would I write in letters of gold the name of my friend and instructor, Dr. C. B. Nottingham. But to return. In this portion of the case is another department for pills or powders, with a closely fitting lid or cover. This completes the description of this most convenient little pocket case, and contains everything needed for ordinary cases of emergency in a general practice.

The Messrs. Tieman, (Geo. Tieman & Co., 67 Chatham street,) of New York, are the makers of this case, together with the pocket thermometer and hypodermic syringe, and extra small points or needles I have referred to, and they have seen proper to call it the "Dr. Greene's Pocket Case and Hypodermic Syringe," by which name it can be ordered at a cost of fifteen dollars. These extra small needles will fit any hypodermic syringe of their make, and I know physicians will discard the larger ones now in use after once trying the former, for their introduction is positively painless when kept in good order. I have, perhaps, written more than is prudent upon the subject of hypodermic medicine, but in all probability this will be my last paper on that or any other subject in medicine, as I am on the eve of retiring from the laborious and perplexing duties of the practice of medicine to the far more agreeable and pleasant pursuits of agriculture. I will embrace this opportunity of thanking my brethren of the medical press for the many acts of courtesy and kindness always extended me in my humble contributions to their medical journals, and while my mind and time will be absorbed in another direction, I shall often think of them in their laborious efforts to build up a high standard of medical literature, and always rejoice with them in their success, and weep with them in their disaster; and may God speed you in all your laudable undertakings, and abundantly reward you for every noble exertion.

## Figures and Tables

**Figure f1:**